# Ten-year clinical and radiographic results of 1000 cementless Oxford unicompartmental knee replacements

**DOI:** 10.1007/s00167-019-05544-w

**Published:** 2019-06-17

**Authors:** Hasan R. Mohammad, James A. Kennedy, Stephen J. Mellon, Andrew Judge, Christopher A. Dodd, David W. Murray

**Affiliations:** 1 Nuffield Department of Orthopaedics, Rheumatology and Musculoskeletal Sciences, University of Oxford, Oxford, UK; 2 Oxford University Hospitals NHS Foundation Trust, Nuffield Orthopaedic Centre, Oxford, UK

**Keywords:** Cementless fixation, Long-term outcomes, Unicompartmental knee replacement

## Abstract

**Purpose:**

Unicompartmental knee replacement (UKR) has substantial benefits over total knee replacement (TKR) but has higher revision rates. The cementless Oxford UKR was introduced to address this but there are concerns about fixation and tibial plateau fractures. The first long-term study of the device with clinical and radiographic outcomes is reported.

**Methods:**

The first 1000 medial cementless Oxford UKR were prospectively identified and followed up by independent physiotherapists. Survival was calculated using the endpoints reoperation, revision, revision to TKR, major revision requiring revision TKR components and patient mortality. The Oxford Knee Score (OKS), Tegner Activity Score and American Knee Society Score (AKSS) were recorded and radiographs analysed.

**Results:**

The ten year survival was 96.6% (CI 94.8–97.8), 97.5% (CI 95.7–98.5), 98.9% (CI 97.7–99.4) and 99.6% (CI 98.8–99.9) using reoperation, revision, revision to TKR and major revision as the endpoint, respectively. Commonest causes for revision were bearing dislocation (*n* = 7, 0.7%), disease progression (*n* = 4, 0.4%) and pain (*n* = 2, 0.2%). There was one lateral tibial plateau fracture and one femoral component loosening. At 10 years, the mean OKS was 41.2 (SD 9.8), Tegner 2.8 (SD 1.3), AKSS-O 89.1 (SD 13.0) and AKSS-F 80.4 (SD 14.6). There were no pathological radiolucencies or complete radiolucent lines. There were no implant-related deaths.

**Conclusions:**

The cementless Oxford UKR is a safe procedure with excellent long-term clinical results. Our results suggest that reliable fixation was achieved with only one (0.1%) revision for loosening (femoral), no radiographic evidence of loosening in the remaining cases and no fractures related to implantation.

**Level of evidence:**

III.

## Introduction

The two main established treatments for end stage medial compartment osteoarthritis are total knee replacement (TKR) and unicompartmental knee replacement (UKR) [26]. UKR has substantial benefits over TKR including a faster recovery, better functional outcomes and cost effectiveness but has higher revision rates [3, 16, 28].

The most commonly used UKR is the Oxford UKR (Zimmer Biomet, Swindon, UK) [26]. The cemented version was introduced in 1998, and is implanted through a minimally invasive approach [7]. The most common causes for revision by the National registers are aseptic loosening and pain [1, 26]. The cementless UKR was introduced in an attempt to improve fixation. Randomised controlled trials (RCTs) have demonstrated a reduced incidence of radiolucencies, shorter surgery times and similar functional outcomes with cementless compared to cemented Oxford UKR [14, 21]. However, these RCTs are limited by sample size and follow-up period. There is now emerging evidence from the New Zealand Joint Registry that the revision rate of the cementless Oxford UKR is significantly less than the cemented version [25]. However, despite this, there is a concern that, in the long term, there may be an increased risk of loosening or possibly a worse functional outcome with the cementless. There are also reports that the cementless is more prone to tibial plateau fractures [24].

This study reports the long-term clinical and radiographic results of the first 1000 cementless UKR performed by two designer surgeons. Previous studies [2, 5, 18] have shown, provided the recommended indications and surgical technique are adhered to, similar results are achieved by designer and non-designer surgeons. Therefore, the results of the study should be generalisable to all surgeons using the recommended indications and techniques [10].

## Methods

Between June 2004 and November 2017, 1000 medial cementless Oxford UKRs were performed through a minimally invasive approach by two designer surgeons (DWM, CAFD) for the recommended clinical indications. Patients were prospectively recruited and independently followed up by research physiotherapists, with clinical assessments at 1, 5, 7 and 10 years post operatively. During the study, 33 knees withdrew from regular follow-up; 22 knees from patients with poor health, 4 knees from patients going abroad and 7 knees from patients requesting to leave the study. However none of these knees were revised or reoperated prior to their withdrawl.

The indications were based on pathoanatomy with the majority being for anteromedial osteoarthritis (AMOA) and a few with medial avascular necrosis. In AMOA, there should be bone on bone medial arthritis, functionally intact anterior cruciate ligament and full thickness cartilage in the weight bearing part of the lateral compartment. Age, activity, body mass index, the site of pain and the state of the patellofemoral joint were not considered contraindications. As a result, the surgeons used UKR for more than half of their knee replacements.

For the survival analysis, failure was defined in various ways including reoperation, revision, revision to TKR and major revision involving the use of revision TKR components. Revision was defined as the removal, addition or replacement of any implant component. Reoperation was defined as any surgical intervention to the knee and included manipulations under anaesthesia, arthroscopies and all revisions. Revision to TKR was defined as the removal of the UKR and insertion of a TKR. Major revision was defined as the usage of revision TKR components which included stems, wedges, augments or constraints.

Functional outcomes were assessed using; Oxford Knee Score (OKS), American Knee Society Objective Score (AKSS-O), American Knee Society Functional Score (AKSS-F) and the Tegner Activity Score. The AKSS-O was calculated as previously described without deductions if the post-operative alignment was not neutral, as the Oxford UKR does not aim to achieve neutral alignment like TKR but aims to restore pre-disease alignment [9].

Complications or further surgeries were recorded when they occurred or at each follow-up appointment. Patients who were unable to attend were contacted by post or telephone to obtain the relevant clinical information. Our prospective database is updated in real time by a full-time data manager and data were extracted on 14th August 2018.

**Fig. 1:**
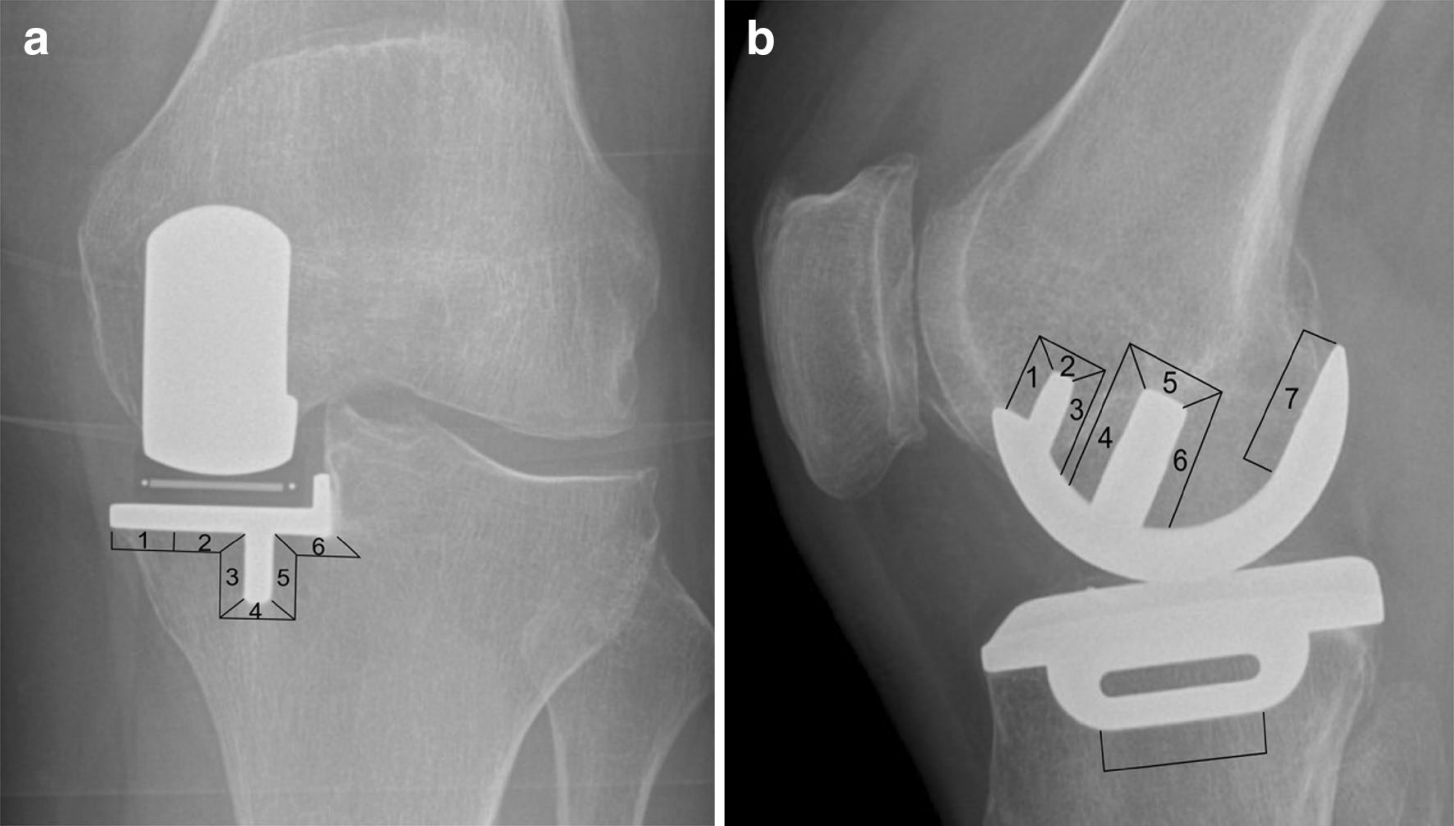
Zones assessed for radiolucencies on radiographs. **a** Anteroposterior radiograph, **b** lateral radiograph

Post-operative well-aligned radiographs were assessed by two independent surgeons (HRM and JK) at a minimum of 5 and 10 years for assessing the presence of radiolucencies and to assess the status of the knee’s lateral compartment. Radiolucencies were categorised as to whether they were pathological, which are poorly defined, progressive and greater than 2 mm thick, or physiological, which are commonly 1 mm thick, well defined with a sclerotic margin and non-progressive [8]. Physiological radiolucencies are otherwise known as radiolucent lines and are not associated with loosening. Radiolucencies around the tibial component were assessed using the anteroposterior radiographs by dividing the region around the tibial tray into six zones [11] (Fig. 1a). Tibial radiolucencies were defined as being complete if all six zones were involved, and partial if under six zones were involved. As in previous studies, the area lateral to the tibial wall was not assessed given this is not deemed to be weight bearing [8, 11]. Radiolucencies around the femoral component were assessed using lateral radiographs by examining the flat region at the posterior femoral condyle and area around the femoral pegs in seven zones (Fig. 1b) [11]. Femoral radiolucencies were defined as complete if all seven zones were involved and partial if under seven zones were involved. The lateral compartment was graded as full thickness cartilage, slight narrowing, major narrowing or bone on bone based on the Kellgren Lawrence grading scale [13].

## Statistics

Continuous variables were described using means, standard deviations (SDs), medians and interquartile ranges (IQRs). Categorical variables were tabulated with absolute frequencies. Continuous data were not normally distributed and therefore, appropriate non-parametric tests were utilised. To analyse differences in functional scores pre-and post-operatively, the Wilcoxon matched sign rank test was utilised. To assess survival at endpoints, the Kaplan–Meier method was utilised. Inter- and intra-observer error for radiographic analysis was performed using Cohen's kappa’s statistic. Statistical analyses were all performed in Stata version 14 (STATA Corp, TX). *P* values of < 0.05 were considered significant, and 95% confidence intervals (CIs) are also reported.

## Ethics and potential conflicts of interest

Ethical approval was sought from the local research ethics committee with formal approval deemed unnecessary under National Health Service research governance arrangements. The study was done in agreement with the ethical standards of the institutional and/or national research committee and with the 1964 Helsinki declaration and its later amendments.

The author or one or more of the authors have received or will receive benefits for personal or professional use from a commercial party related directly or indirectly to the subject of this article. In addition, benefits have been or will be directed to a research fund, foundation, educational institution, or other non-profit organisation with which one or more of the authors are associated.

## Results

Of the 1000 knees, 748 were unilateral and 252 were bilateral, of which 4 knees were simultaneous. There were 536 (54%) male knees and 464 (46%) female knees with a mean age at surgery of 66.2 years (SD 10.0). There were 495 left- and 505 right knees operated on and the mean BMI was 29.2 (SD 5.0). 988 knees had a diagnosis of anteromedial osteoarthritis and 12 had spontaneous osteonecrosis of the knee. All patients satisfied the recommended indications as described by Goodfellow et al. [6]. The mean follow-up (*n* = 1000) was 5.1 years (SD 2.6) with 262 knees having follow-up of 7 years and over, and 29 knees with follow-up of 10 plus years.

**Fig. 2:**
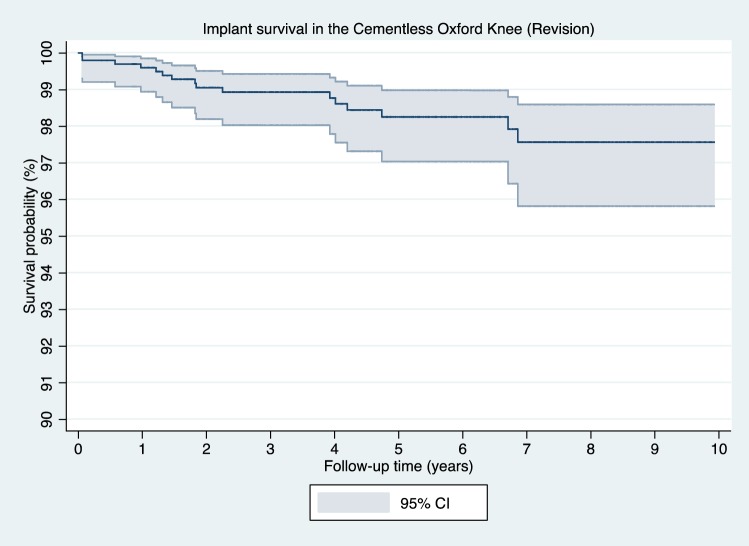
Implant survival based on revision as the endpoint

There were 25 reoperations from the cohort at a mean of 2.6 years (SD 2.6). Using reoperation as an endpoint, the implant survival was 97.3% (CI 95.9–98.2) at 5 years and 96.6% (CI 94.8–97.8) at 10 years (Table 1). From the 25 reoperations, 18 met the definition of implant revisions at mean 3.0 years (SD 2.8). Using revision as an endpoint, the implant survival was 98.1% (CI 96.9–98.9) at 5 years and 97.5% (CI 95.7–98.5) at 10 years (Table 1, Fig. 2). The commonest reasons for revision were bearing dislocation (7 knees, 0.7%), disease progression in the lateral compartment (4 knees, 0.4%) and pain (2 knees, 0.2%) (Table 2).

**Table 1:** Implant survival at each year for revision and reoperation

Follow-up (years)	Number at risk	Revision survival (95% CI)	Reoperation survival (95% CI)
1	976	99.6 (98.9–99.9)	99.3 (98.5–99.7)
2	845	99.1 (98.2–99.5)	98.7 (97.7–99.2)
3	737	98.8 (97.9–99.3)	98.2 (97.1–98.9)
4	614	98.7 (97.6–99.2)	98.0 (96.9–98.8)
5	494	98.1 (96.9–98.9)	97.3 (95.9–98.2)
6	373	98.1 (96.9–98.9)	97.3 (95.9–98.2)
7	262	97.5 (95.7–98.5)	96.6 (94.8–97.8)
8	137	97.5 (95.7–98.5)	96.6 (94.8–97.8)
9	65	97.5 (95.7–98.5)	96.6 (94.8–97.8)
10	29	97.5 (95.7–98.5)	96.6 (94.8–97.8)

**Table 2:** Summary of the first re-operations and revisions patients experienced in the 1000-patient cohort

Indication	Number of knees	Surgical procedure conducted	Mean time from initial surgery (years)	Number of reoperations	Number of revisions
Bearing dislocation	7	7 Bearing exchange	2.9 (SD 3.4)	7	7
Disease progression	4	2 TKRs 2 Lateral UKR	4.8 (SD 1.4)	4	4
Pain	5	2 Arthroscopies 1 Tibial revision 1 TKR 1 Aspiration and MUA	1.9 (SD 1.8)	5	2
Infection	3	1 Arthroscopy and washout 2 debridement, washout and bearing exchange.	0.04 (SD 0.02)	3	2
Avascular necrosis	1	1 Lateral UKR	6.7	1	1
Femoral component loosening	1	1 Cemented femoral component	0.6	1	1
Lateral meniscal tear	1	1 Arthroscopy	4.8	1	0
Wound dehiscence	1	1 Debridement and primary closure	0.2	1	0
Loose body	1	1 Arthroscopy and arthrotomy	2.1	1	0
Tibial plateau fracture (lateral)	1	1 TKR	2.2	1	1
Totals				25	18

Using revision to TKR as the endpoint, survival at both 5 and 10 years was 98.9% (CI 97.7–99.4). There was a total of eight revisions to TKR (4 of which were after a failed initial non TKR revision) at a mean time of 3.0 years (SD 1.6); two knees for lateral compartment arthritis, one knee for pain, one knee for a lateral tibial plateau fracture following a fall, two for infection and two for recurrent bearing dislocations. The revision for pain was done in a different institution and the source of pain is not clear. However it was noted at both the primary and the revision procedure, that there was some patellofemoral osteoarthritis.

Using major revision as the endpoint, survival at both 5 and 10 years was 99.6% (CI 98.8–99.9). There were a total of three major revisions at a mean of 2.4 years (SD 1.6). One knee was converted to a TKR with a stemmed tibial implant following a lateral tibial plateau fracture after a fall that did not improve with non-operative management, one knee was converted to TKR with tibial stem for lateral disease progression and one knee had a deep infection requiring a two-stage revision with a tibial stem and augment.

**Fig. 3:**
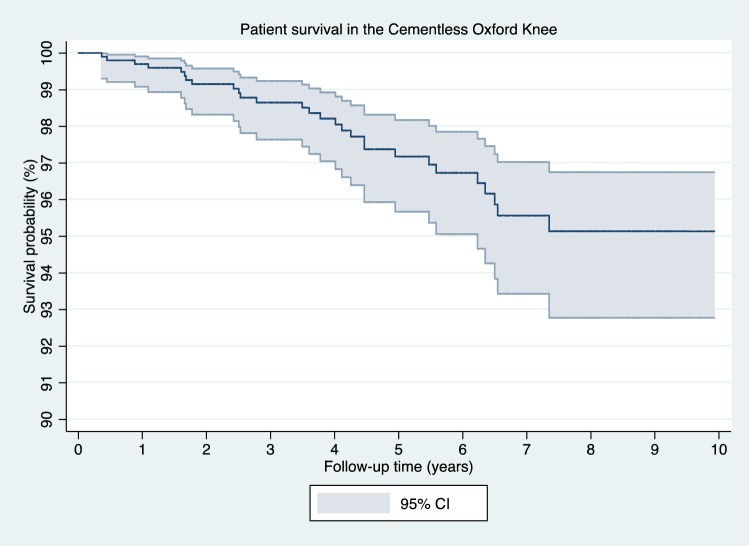
Patient survival in the cementless cohort

There were 28 deaths during the duration of the study at a mean 3.6 years (SD 2.0). This corresponds to a patient survival of 97.2% (CI 95.7–98.2) at 5 years and 95.1% (CI 92.8–96.7) at 10 years (Fig. 3). No death was related to the implant and no patient died within 130 days of surgery.

The most recent outcome scores (OKS, Tegner, AKSS-O and AKSS-F) were all significantly better (*p* < 0.001) than their respective preoperative scores. At 1, 5, 7 and 10 years, 68, 69, 74 and 73%, respectively, had an excellent OKS (> 41) with only 3.6% having a worse OKS post-operatively than preoperatively. Outcome scores at different time points are summarised in Table 3. The most recent mean and median flexion angle was 127.4° (SD 10.3, range 80–152) and 128.5° (IQR 14). The most recent mean and median extension angle was − 2.71° (SD 4.4, range − 20 to 10) and − 2° (IQR 5).

**Table 3:** Mean and median patient-reported outcome measures preoperatively and at 1-, 5-, 7- and 10-year follow-up

PROM score	Time point				
	Preop	1 year	5 years	7 years	10 years
OKS	25.1 (SD 8.5) 25.0 (IQR 12.0)	41.6 (SD 7.7) 45.0 (IQR 8.0)	42.0 (SD 7.3) 45.0 (IQR 8.0)	42.3 (SD 7.4) 45 (IQR 6.0)	41.2 (SD 9.8) 45.0 (IQR 6.0)
Tegner	2.4 (SD 1.1) 2 (IQR 1.0)	3.0 (SD 1.3) 3.0 (IQR 2.0)	3.1 (SD 1.5) 3.0 (IQR 2.0)	2.9 (SD 1.5) 3.0 (IQR 1.0)	2.8 (SD 1.3) 3.0 (IQR 1.0)
AKSS-O	60.2 (SD 15.4) 60.0 (IQR 21.0)	91.7 (SD 11.4) 95.0 (IQR 7.0)	93.6 (SD 9.0) 95.0 (IQR 8.0)	88.5 (SD 16.7) 94.0 (IQR 9.0)	89.1 (SD 13.0) 93.0 (IQR 7.0)
AKSS-F	70.8 (SD 16.8) 70.0 (IQR 20.0)	82.3 (SD 17.7) 80.0 (IQR 30.0)	83.6 (SD 19.4) 90.0 (IQR 25.0)	83.3 (SD 18.2) 90.0 (IQR 30.0)	80.4 (SD 14.6) 80.0 (IQR 20.0)

The inter- and intra-observer error for radiolucency analysis was kappa = 0.80 (*p* < 0.001) and kappa 0.90 = (*p* < 0.001) which is acceptable and similar to those previously reported for this type of analysis [15]. Both the inter- and intra-observer errors for lateral compartment radiographic grading were kappa = 0.85 (*p* < 0.001).

**Fig. 4:**
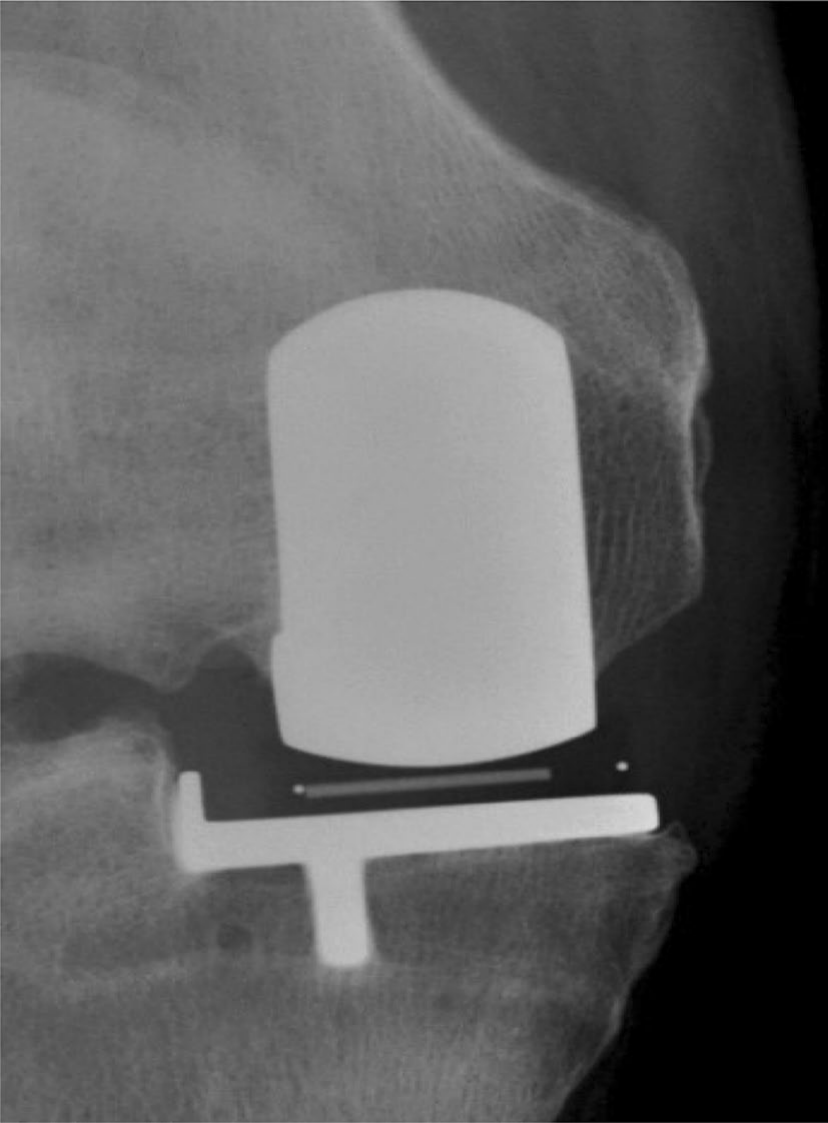
Worst tibial radiolucency observed in the cohort

348 radiographs (70%) were available for patients with a minimum follow-up of 5 years. On these radiographs, there were no tibial or femoral pathological radiolucencies. There were 0 (0%) complete and 45 (13%) partial tibial radiolucent lines (Fig. 4). The sites for tibial radiolucencies were zone 6 (*n* = 36), zone 5 (*n* = 11), zone 3 (*n* = 9), zone 4 (*n* = 1) and zone 1 (*n* = 1). There were 0 (0%) complete femoral radiolucent lines and 4 (1.1%) partial femoral radiolucent lines with one in zone 1, 3, 4 and 6 respectively. Regarding the lateral compartment assessment, there were 309 (88.7%) knees with full thickness compartments, 24 (6.9%) knees showing slight narrowing, 12 (3.4%) knees showing marked narrowing and 3 (0.9%) knees demonstrating evidence of bone on bone contact.

Twenty-two radiographs (76%) were available for patients with follow-up of 10 years and over, and none showed pathological radiolucencies. There were 2 (9%) partial tibial radiolucent lines both of which were in zone 6 and no (0%) complete tibial radiolucent lines. There was one (4.5%) partial femoral radiolucent line in zone 4 and no (0%) complete femoral radiolucent lines. Regarding the lateral compartment, there were 20 (91%) knees that had full thickness compartments, one (4.5%) with marked narrowing and one (4.5%) with evidence of bone on bone contact.

## Discussion

The most important finding of our study is that excellent mid- to long-term clinical and radiographic outcomes can be achieved using the cementless Oxford UKR. Overall, the outcomes were similar to those reported in an almost identical study of the cemented version [22]. However, the cementless does appear to have some advantages, for example, there was a lower incidence of radiolucent lines suggesting improved fixation and a lower incidence of revision for pain. The indications used for the cementless were the same as the recommended indications used for the cemented, which are satisfied in about half of the patients needing primary knee replacement [27, 28]. Although our results are from designer surgeons they are generalisable given previous studies and a meta-analysis have shown that similar results can be achieved by designer and non-designer surgeons providing they use the recommended indications and techniques [2, 5, 18].

Traditionally, the outcome of knee replacement has been assessed based on revision rate. When revision is considered to be a failure, the 10-year survival of the cementless UKR is 97.5% (CI 95.7–98.5). This is similar to the survival of 96% (CI 92.5–99.5) previously reported of the cemented Oxford UKR [22]. It is also similar to the 96% survival reported in a meta-analysis of 20,873 TKRs [17]. However, if all reoperations, including revision, are considered to be failures, then there are advantages for the cementless UKR compared to TKR: the ten survival of the cementless UKR is 96.6% (CI 94.8–97.8) which is better than similar studies for TKR (86.7%) [19]. Revisions of TKR tend to be complex procedures with almost all requiring revision TKR components with stems and wedges. If revision requiring revision TKR components is considered to be failure, then the cementless UKR also does well with a 10-year survival of 99.6% (CI 98.8–99.9%). The patient mortality was low with no deaths related to the implantation and a 10-year patient survival of 95.1% which is better than that reported for TKRs, although this may in part be because TKR patients tend to be older [16].

The primary patient-reported outcome in this study was the OKS. The mean post-operative OKS did not change appreciably with time and at 1, 5, 7 and 10 years, it was between 41.2 and 42.3. At each time point the mean score classified as excellent (> 41) according to the OKS criteria and the median OKS at each postoperative time point was 45. These scores are similar to those in the previously reported cemented cohort which reports post-operative mean OKS scores of 40 [20, 22]. The post-operative OKS appears better than those in similar TKR studies (at 1, 5, 7 and 10 years, ranging from 33.5 to 35) [19]. From a patient’s perspective, they would want to avoid being worse post-operatively than pre-operatively. For the cementless UKR, 3.6% had a worse post-operative score than pre-operative which is half of that reported for TKR [12]. Better functional outcomes in UKR compared to TKR have also been reported in a recent large meta-analysis [28].

The most common causes for revision were bearing dislocation (*n* = 7, 0.7%), disease progression (*n* = 4, 0.4%) and pain (*n* = 2, 0.2%). These were also the commonest reasons for revision reported in the cemented 1000 cohort, except in the cemented cohort pain (*n* = 10, 1%) was the leading cause of revision followed by disease progression (0.7%, *n* = 9), and then bearing dislocation (0.6%, *n* = 6) [22]. The rate of revision for pain was lower for cementless than cemented. In the cementless study, six out of the seven bearing dislocations were successfully treated with a bearing replacement. It is generally believed that following UKR, lateral compartment arthritis will inevitably progress leading to revision. Our results show that revision for disease progression is rare with only four knees (0.4%) requiring revision for lateral progression (two with TKR and two with lateral UKR), although at 10 years 9% (2/22) had radiographic evidence of lateral arthritis.

There were three cases (0.3%) of infection: one was treated by arthroscopy and washout, and two by debridement, washout and bearing exchange. The later two cases were subsequently converted to a TKR (1 single-stage TKR and 1 two-stage TKR). The cemented cohort had six cases (0.6%) of infection all of which were treated with a two-stage TKR [22].

The main theoretical concern about changing from cemented to cementless fixation is that there might be an increased risk of aseptic loosening [4]. This did not occur and the incidence of revision for cementless loosening (0.1%) was the same as in our similar cemented cohort [22]. In the cementless study, there were no revisions for tibial component loosening and only one case (0.1%) of femoral component loosening, which was treated by replacement with a cemented femoral component. This component was probably not securely fixed at the time of surgery because the hole for the main peg on the component which provides primary fixation had been damaged. It is likely that this was damaged by an instrument designed to remove a collar of bone that is no longer recommended for cementless fixation. In addition, there was no radiographic evidence of tibial or femoral loosening, with no pathological radiolucencies. Furthermore, there were no complete radiolucent lines (physiological radiolucencies) and only 13% of tibial components and 1% of femoral components had had partial radiolucent lines. In contrast around the cemented components, about one-third of the tibial components have complete radiolucent lines [8]. Although these are not indicative of loosening, it does suggest that the fixation of the cementless components is better than cemented and that if patients have pain, cementless components are less likely to be revised than cemented.

Peri-prosthetic fractures are a recognised complication of UKR with a multifactorial aetiology [23]. There is a concern that impaction of press fit cementless components may increase the risk of fracture. Furthermore, there are anecdotal reports of an increased incidence of tibial plateau fracture following cementless fixation and cadaveric work suggests that these fractures are more likely to occur with cementless than cemented UKR [24]. There was only one fracture in this study. It was a lateral tibial plateau fracture following a traumatic fall that occurred over two years after the surgery. It was, therefore, not related to the implant or its implantation. This, therefore, suggests that the risk of implant-related fracture is likely to be very small with careful adherence to the recommended surgical technique.

In general, the reported UKR revision rates in Registries [1, 25, 26] is higher than in large cohort studies like this one. This is primarily because most surgeons reporting their results to registries do small numbers of UKR and only use UKR for a small proportion of their knee replacements, which suggests there may be poor adherence to the recommended indications and techniques. However, recent reports from the New Zealand Registry have shown that the revision rate of the cementless Oxford is about half that of the cemented [25]. This may be because of the improved fixation or it may be because more experienced surgeons are using the cementless implant. Further study is needed.

The main strengths of this study are that it is a well-documented, large prospective consecutive series of 1000 cementless Oxford UKRs using the recommended indications and techniques with independent physiotherapy follow-up providing survival, subjective and objective outcomes and radiographic data. The main weaknesses are that there was no comparator arm in the study and hence, our results have been compared to a similar published cemented UKR cohort [22], TKR cohort [19] and meta-analyses [17, 18] in the literature. Additionally, this is a single-centre study with patients from one region within the UK.

## Conclusions

Excellent long-term functional outcomes, survival and radiographic results can be achieved with the cementless Oxford UKR, when used for the recommended indications. The main concerns with cementless fixation are that it may lead to increased loosening rates or to fractures from impaction of press fit components. These results suggest that reliable fixation was achieved with only one (0.1%) revision for loosening (femoral), no radiographic evidence of loosening in the remaining cases and no fractures related to the implant’s implantation.

## Abbreviations

AKSS-F: American Knee Society Functional Score
AKSS-O: American Knee Society Objective Score
AMOA: Anteromedial osteoarthritis
CI: Confidence intervals
IQR: Interquartile range
OKS: Oxford Knee Score
RCT: Randomised controlled trial
SD: Standard deviation
TKR: Total knee replacement
UKR: Unicompartmental knee replacement
